# Cortical Thickness Alterations in Patients With Tinnitus Before and After Sound Therapy: A Surface-Based Morphometry Study

**DOI:** 10.3389/fnins.2021.633364

**Published:** 2021-03-05

**Authors:** Xuan Wei, Han Lv, Qian Chen, Zhaodi Wang, Chunli Liu, Pengfei Zhao, Shusheng Gong, Zhenghan Yang, Zhenchang Wang

**Affiliations:** ^1^Department of Radiology, Beijing Friendship Hospital, Capital Medical University, Beijing, China; ^2^Department of Otolaryngology Head and Neck Surgery, Beijing Friendship Hospital, Capital Medical University, Beijing, China

**Keywords:** tinnitus, sound therapy, surface-based morphometry, thickness, structural magnetic resonance imaging

## Abstract

This study aimed to explore brain surface-based morphometry cortical thickness changes in patients with idiopathic tinnitus before and after 24 weeks of sound therapy. In this prospective observational study, we recruited 33 tinnitus patients who had undergone 24 weeks of sound therapy and 26 matched healthy controls. For the two groups of subjects, a 3D-BRAVO pulse sequence was acquired both at baseline and at the 24th week. Structural image data preprocessing was performed using the DPABISurf toolbox. The Tinnitus Handicap Inventory (THI) score was assessed to determine the severity of tinnitus before and after treatment. Two-way mixed-model analysis of variance (ANOVA) and Pearson’s correlation analysis were used in the statistical analysis. Student–Newman–Keuls (SNK) tests were used in the *post hoc* analysis. Significantly lower cortical thickness was found in the left somatosensory and motor cortex (SMC), left posterior cingulate cortex (PCC), and right orbital and polar frontal cortex (OPFC) of the participants in the tinnitus group at baseline than in the participants in the HC group at baseline and after 24 weeks; in the tinnitus group, significantly higher cortical thickness was found after the 24 weeks sound therapy in comparison to the baseline in the left SMC, bilateral superior parietal cortex (SPC), left inferior parietal cortex (IPC), left PCC, and right OPFC. In the HC group, no statistically significant difference in cortical thickness was found after the 24 weeks treatment in comparison to the baseline in the bilateral SMC, bilateral SPC, left IPC, left PCC, or right OPFC. The changes in cortical thickness before and after sound therapy can provide certain reference values for clinical tinnitus treatment. These brain regions could serve as potential targets for neuroimaging.

## Introduction

Tinnitus is characterized by the ability to hear sound in the ears or head without any external stimulation (Wegger et al., 2017; Bauer, 2018). The adult prevalence rate is 10–15% (Henry et al., 2020), and the prevalence rate in elderly individuals is approximately 32.0% (Chang et al., 2019). Tinnitus patients exhibit varying degrees of hearing impairment (Cheng et al., 2020). In addition to hearing impairments, annoying tinnitus is often associated with sleep disorders, depression, anxiety, and other mental illnesses and severely impairs patients’ quality of life (Reynolds et al., 2004; Langguth et al., 2013; Zeman et al., 2014). In turn, these disorders can further exacerbate tinnitus (Bhatt et al., 2017).

One promising avenue to help in the search for therapeutic markers is neuroimaging biomarker research (Liu et al., 2020). Tinnitus was indicated to be a disease characterized by abnormal resting-state functional magnetic resonance imaging (rs-fMRI) in our previous research (Han et al., 2015; Lv et al.,2016a,b, 2018b). We also used various network-based approaches, such as graph-theoretical methods and tract-based spatial statistics (TBSS), to investigate associations with clinical variables (Lv et al., 2018a; Han et al., 2019b; Chen et al., 2020b). The brain regions involved include sound detection regions, such as the insula and hippocampus (van der Loo et al., 2011; Hofmeier et al., 2018), and auditory and non-auditory brain regions (Vanneste and De Ridder, 2012), such as the parahippocampal gyrus (Vanneste et al., 2011), posterior cingulate cortex (PCC) (Vanneste et al., 2010), and anterior cingulate cortex (Dirk et al., 2011). Previous studies have also shown that tinnitus can cause significant changes in brain function and structure, which were closely related to the clinical manifestations of patients (Ryu et al., 2016; Schmidt et al., 2017; Han et al., 2019a).

In recent years, an increasing number of studies have begun to focus on the microstructure of the brain. Our previous studies reported brain structural and white matter (WM) microstructural reorganization in the early stage of tinnitus (Chen et al.,2020a,b). Therefore, it is very important to fully understand the abnormal brain structure associated with tinnitus. Microstructural changes in the brain have also been reported in some tinnitus studies (Tae et al., 2018; Besteher et al., 2019). In these studies, the measurements of the brain structure’s microstructural changes have usually focused on two methods: voxel-based morphometry (VBM) and surface-based morphometry (SBM). Some studies have reported microstructural changes in the brains of tinnitus patients with VBM (Husain et al., 2011), which can quantitatively detect the volume of brain tissue at the voxel level (Ashburner and Friston, 2000). Our previous study also applied VBM and found significantly decreased gray matter (GM) volume in the left thalamus, right thalamus, and cochlear nucleus in tinnitus patients before sound therapy (baseline) compared to the healthy control (HC) group (Wei et al., 2020b). By contrast, SBM focuses on cortical structural characteristics. This important difference between SBM and VBM is how these morphological methods identify different brain regions. In recent years, individual studies have reported that the results of VBM cannot be directly compared with the results of SBM because the latter is a more innovative method in studying the structural characteristics associated with tinnitus (Leaver et al., 2012). The differences in VBM and SBM findings are due to the targeted structures using each technique and the variability in brain morphometry techniques (Adjamian et al., 2014). Unlike VBM analyses that mainly focus on subtle distinctions in volume (Frydman et al., 2016), SBM focuses on cortical structural characteristics such as thickness, volume, surface area, and curvature of the cerebral cortex (Khan et al., 2014). Meyer et al. (2016) revealed that cortical thickness differentially correlated with tinnitus distress and duration. Yoo et al. (2016) found that the cortical thickness changes related to hearing loss overlapped with those related to normal aging. However, to date, changes in cortical thickness have not been examined in the evaluation of the treatment efficacy of tinnitus.

Having a basic understanding of brain function and anatomical changes is necessary to effectively treat tinnitus. Many treatment modalities have been applied to tinnitus patients, such as repetitive transcranial magnetic stimulation (rTMS) (Poeppl et al., 2018), drug therapy (Zenner et al., 2017), tinnitus counseling and cognitive behavioral therapy (CBT) (Langguth et al., 2013), hearing aids (Yakunina et al., 2019), cochlear implants (Olze, 2015), and tinnitus retraining therapy (Lee et al., 2019). A previous study applied music therapy and observed changes in the GM volume of tinnitus patients; after the Heidelberg model of music therapy intervention, GM volume in the medial superior frontal areas, auditory cortex, and precuneus increased in acute tinnitus patients, accompanied by significantly decreased tinnitus-related distress (Krick et al., 2015). In recent years, narrowband noise sound therapy has been one of the most commonly used methods for the treatment of tinnitus (Henry et al., 2002). In our study, narrowband sound was defined as a sound center frequency of 400 Hz or higher: the 1/3 octave band is the narrowest and the 1/2 octave band is the widest. This narrowband noise sound therapy is often used clinically to improve short-term effects, and most current therapies are modified using only one characteristic of the individual and/or their tinnitus (Searchfield et al., 2017). Our previous studies demonstrated functional changes in the brain with this sound therapy (Han et al., 2019a,b). However, there are few related reports on surface-based morphological changes, especially changes in cortical thickness, before and after narrowband noise sound therapy.

In this study, tinnitus patients who underwent 24 weeks of narrowband noise sound therapy were enrolled. SBM was applied to analyze the anatomical changes in the brain in patients before and after sound therapy as well as to acquire data from HCs at baseline and at 24 weeks to explore morphological feature alterations. A previous study reported that the left auditory cortex and subcallosal anterior cingulate show correlation between tinnitus duration and cortical thickness (Meyer et al., 2016). However, very few studies have paid attention to the effect of sound therapy on the thickness of the cortex and on which brain regions are more involved. Therefore, we hypothesize that the brain regions associated with tinnitus in SBM may show cortical thickness increase after sound therapy. We hypothesized that sound therapy can return the brain structure to a relatively normal range. Hence, we attempt to provide deeper insight into the changes in the brain after long-term treatment for tinnitus from a surface-based morphological and neuroanatomical perspective.

## Materials and Methods

### Standard Protocol Approval, Registration, and Patient Consent

This research involved human participants. All authors have declared that this research was approved by the institutional review board (IRB). This study was approved by the Ethics Committees of our Research Institution (Beijing Friendship Hospital, Capital Medical University, 2016-P2-012). Written informed consent was obtained from all study subjects.

### Subjects

All patients and healthy volunteers were recruited in our institution. In this study, 33 patients with idiopathic tinnitus were enrolled. Two patients had right laterality and five had left laterality; the rest had bilateral laterality. The tinnitus sound was described as a persistent, low- or high-pitched sound in one or both ears. A clinically considered high-frequency sound is more than 4 kHz. The inclusion criteria were as follows: (1) age of 18 to 65 years; (2) right handedness; (3) tinnitus duration ranging from 6 to 48 months; (4) no significant hearing loss (hearing thresholds ≥ 25 dB HL at frequencies of 0.250, 0.500, 1, 2, 3, 4, 6, and 8 kHz determined by pure tone audiometry (PTA) examination; and (5) willingness to receive sound therapy for 24 weeks and then return for re-examination after treatment. The exclusion criteria included: (1) other kinds of tinnitus (such as pulsatile tinnitus), Meniere’s disease, sudden deafness, or otosclerosis; (2) neurological signs and/or a history of neurological disease; (3) current chronic medical illness; (4) a history of head trauma; (5) cardiovascular, pulmonary, or systemic disease; (6) claustrophobia experienced during the MRI simulator session; and (7) inability to pitch-match their tinnitus. Twenty-six age-, sex-, education-, and handedness-matched HC subjects were enrolled as controls. None of the HCs had suffered from tinnitus in the past year. Other exclusion criteria were the same as previously described. The characteristics of the subjects are presented in Table 1.

**TABLE 1:** Demographic and clinical characteristics of participants.

Characteristics	Tinnitus patients (baseline, *n* = 33)	Tinnitus patients (24th week, *n* = 33)	Healthy controls (baseline, *n* = 26)	Healthy controls (24th week, *n* = 26)	*P*-values
Age (years, $\bar{x}$ ± s)	48.2 ± 12.4		47.3 ± 9.6		0.745^*a*^
Gender (male/female)	23/10		15/11		>0.99^*b*^
Handedness	33 right-handed		26 right-handed		>0.99^*a*^
Tinnitus duration (months)	≥6 and ≤48		NA		NA
Tinnitus pitch	250–8,000 Hz				NA
THI score	52.5 ± 44.3	37.3 ± 20.9	NA	NA	0.011^*c*^
ΔTHI score	15.3 ± 32.8	NA	NA	NA	NA
Laterality	2 right, 5 left, 26 bilateral				

*Data are presented as mean ± SD for all variables except gender. THI, Tinnitus Handicap Inventory; ΔTHI, THIpre – THIpost; NA, not applicable. ^*a*^Two-sample t-tests. ^*b*^Chi-square test. ^*c*^Paired-samples t-tests.*

### Sound Therapy and Clinical Evaluation

We used the TinniTest^®^ (TTS-1000A, China) tinnitus comprehensive diagnosis and treatment instrument for psychoacoustic testing and the SpeechEasy eMasker^®^ (Micro-DSP Technology Co., Ltd, China) for narrowband noise sound therapy. We advised patients to use it in a quiet environment to achieve the best therapeutic effect. All of the enrolled tinnitus patients were examined for tinnitus loudness matching (L = loudness of tinnitus), pitch matching (Tf = tinnitus frequency), minimum masking level, and residual inhibition to characterize the tinnitus and prepare for treatment. Narrowband noise sound therapy was administered to the participants in the tinnitus group for 24 weeks, three times a day for 20 min each time. For each tinnitus patient, the loudness of sound for treatment was set as L-5 dB. The bandwidth is changed according to the center frequency, and the bandwidth is 1/3 octave (for example, Tf = 3 kHz, low cut = 3 kHz × 2^–1/6^, high cut = 3 kHz × 2^1/6^).

We also asked the patients to complete the Tinnitus Handicap Inventory (THI) to assess the severity of tinnitus before and after treatment. The primary outcome of this prospective study was the change in THI score after treatment. A reduction of at least 16 points in the THI score was considered effective treatment (Newman et al., 1996). The HC group was not given any kind of sound during the study.

### Data Acquisition

For each patient, to evaluate the change in brain activity following treatment, structural MRI data were collected at baseline and at the end of therapy (24th week). The HC group was also scanned at baseline as well as at the 24th week. Images were obtained using a 3.0 T MRI system (Prisma, Siemens, Erlangen, Germany) with a 64-channel phase-array head coil. All imaging studies were performed at the Medical Imaging Research Center of our hospital. Parallel imaging was employed for data acquisition. High-resolution three-dimensional (3D) structural T1-weighted (T1w) images were acquired using a 3D magnetization-prepared rapid gradient echo (MP-RAGE) sequence with the following parameters: repetition time (TR) = 2,530 ms, echo time (TE) = 2.98 ms, inversion time (TI) = 1,100 ms, flip angle (FA) = 7°, number of slices = 192, slice thickness = 1 mm, bandwidth = 240 Hz/Px, field of view (FOV) = 256 mm × 256 mm, and matrix = 256 × 256, resulting in an isotropic voxel size of 1 mm × 1 mm × 1 mm.

### Anatomical Data Preprocessing

The results included in this manuscript come from the preprocessing performed by DPABISurf using fMRIPrep 20.0.5 (Esteban et al., 2019), which is based on Nipype 1.4.2 (Gorgolewski et al., 2011). For more details about the pipeline, see the section corresponding to workflows *in* fMRIPrep’s documentation.

The T1w image was corrected for intensity non-uniformity (INU) with N4BiasFieldCorrection (Tustison et al., 2010), distributed with ANTs 2.2.0 (Avants et al., 2008), and used as T1w-reference throughout the workflow. The T1w-reference was then skull-stripped with a *Nipype* implementation of the antsBrainExtraction.sh workflow (from ANTs), using OASIS30ANTs as the target template. Brain tissue segmentation of cerebrospinal fluid (CSF), WM, and GM was performed on the brain-extracted T1w using fast (FSL 5.0.9) (Zhang et al., 2001). Brain surfaces were reconstructed using recon-all (Dale et al., 1999), and the previously estimated brain mask was refined to reconcile ANT-derived and FreeSurfer-derived segmentations of the cortical GM with a custom variation in the method of Mindboggle (Klein et al., 2017). Volume-based spatial normalization to one standard space (MNI152NLin2009cAsym) was performed through non-linear registration with ANTs registration (ANTs 2.2.0) using brain-extracted versions of both the T1w-reference and the T1w template. The following template was selected for spatial normalization: ICBM 152 non-linear asymmetrical template version 2009c (Fonov et al., 2009). A Human Connectome Project (HCP) template is a built-in template in DPABISurf software, and it is used to divide the cortex.

### Statistical Analysis

Demographic data were compared through two-sample *t*-tests and paired two-sample *t*-tests using SPSS 19.0 software (SPSS, Inc., Chicago, IL). *P* < 0.05 were considered statistically significant. Longitudinal changes in THI scores were also analyzed by using paired two-sample *t*-tests.

We used DPABISurf^1^ for preprocessing the neuroimaging statistics. For surface-based cortical thickness data, to determine the group × time interaction effect between the two groups and the two scans, the main effects of group (the tinnitus patient group and the HC group) and time (baseline and 24 weeks follow-up period), two-way mixed model ANOVA and *post hoc* analyses were performed. SBM analyses were conducted using whole-brain analyses. A *P*-value of cortical thickness analysis less than 0.05 (*P* < 0.025 for each hemisphere) was considered statistically significant (Monte Carlo simulation corrected). We looked up tables of *P*-values based on simulations in which a *Z* field was synthesized on the atlas surface. The tables are distributed in DPABISurf. The *P*-value for the cluster is determined by indexing into the table based on the size of the cluster, the threshold used to form the cluster, and an estimate of the global full width/half maximum (FWHM). Clusters are extracted separately for both hemispheres. In *post hoc* analyses, Student–Newman–Keuls (SNK) tests were used for pairwise comparisons. Pearson’s correlation analyses were further conducted to investigate the relationships between the change in cortical thickness and 24 weeks of sound therapy of tinnitus patients (ΔTHI score = THIpre - THIpost). *P* < 0.05 was set as the threshold to determine significance. The cortical thickness results were visualized with DPABISurf (see Footnote 1). Cortical thickness was quantified from the T1w images using DPABISurf V1.4 software. Pearson’s correlation analysis between the THI scores was performed using SPSS 19 software (SPSS, Inc., Chicago, IL).

## Results

### Demographics and Behavioral Outcomes of Study Participants

In this study, we enrolled 33 patients with idiopathic tinnitus, and we applied DPABISurf to analyze the cortical thickness in the brain in this group before and after sound therapy. Concurrently, 26 HC individuals were enrolled. Each group of subjects was age-, sex-, and handedness-matched (Table 1). THI scores were acquired before and after sound therapy. Significant longitudinal decreases in THI scores were observed. The results are summarized in Table 1.

### Statistical Analysis Results

#### Group Differences in Cortical Thickness

**FIGURE 1:**
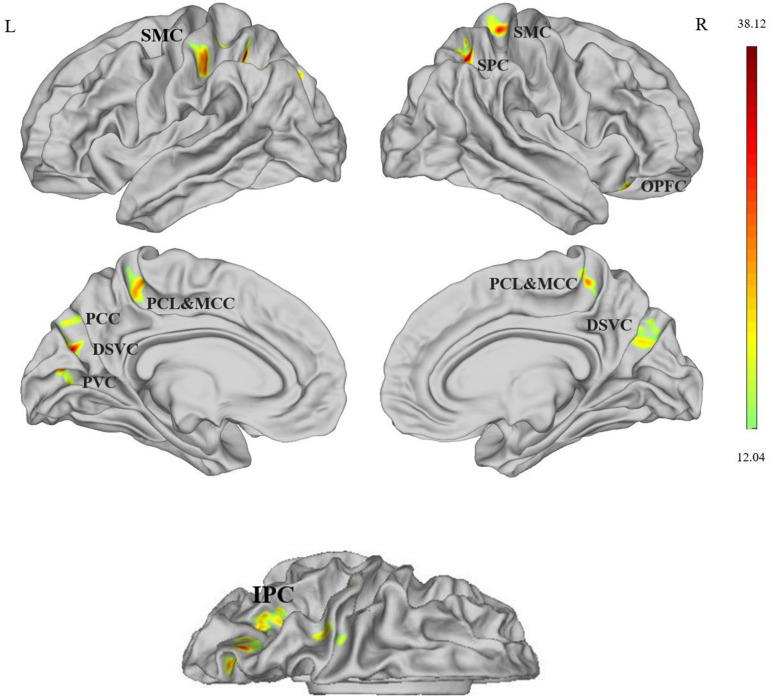
Cortical thickness differences assessed by ANOVA, compared among the four data sets, significant group × time interaction effects between the two groups (tinnitus patients and healthy controls) and two scans (at baseline and at the 24th week) on cortical thickness were observed at the left SMC, bilateral DSVC, bilateral PCL and MCC, bilateral SPC, left IPC, left PCC, left PVC, left PCC, and right OPFC [*P* < 0.05 (*P* < 0.025 for each hemisphere) corrected by Monte Carlo simulation (L, left; R, right)]. Abbreviations: SMC, somatosensory and motor cortex; SPC, superior parietal cortex; PCL and MCC, paracentral lobular and midcingulate cortex; DSVC, dorsal stream visual cortex; PVC, primary visual cortex; PCC, posterior cingulate cortex; OPFC, orbital and polar frontal cortex; IPC, inferior parietal cortex.

As shown in Figure 1 and Table 2, in the comparison among four data sets, significant group × time interaction effects between the two groups (tinnitus patients and HCs) and two scans (at baseline and at the 24th week) on cortical thickness were observed at the bilateral somatosensory and motor cortex (SMC), bilateral dorsal stream visual cortex (DSVC), bilateral paracentral lobular and midcingulate cortex (PCL and MCC), bilateral superior parietal cortex (SPC), left inferior parietal cortex (IPC), left PCC, left primary visual cortex (PVC), and right orbital and polar frontal cortex (OPFC) [*P* < 0.05 (*P* < 0.025 for each hemisphere) corrected by Monte Carlo simulation (L, left; R, right)].

**TABLE 2:** Difference in cortical thickness of the left and right hemispheres between the two groups (tinnitus patients and healthy controls) and two scans (at baseline and at the 24th week).

Brain regions		HCP	Cluster size (mm)	Coordinates MNI			Peak *F* score
				x	y	z	
Somatosensory and motor cortex	L	52	227	–38	–29	40	26.95
	R	9	117	20	–29	53	26.65
Dorsal stream visual cortex	L	17	210	–20	–74	38	30.54
	R	16	113	25	–71	35	19.31
Inferior parietal cortex	L	145	322	–29	–66	40	23.23
Primary visual cortex	L	1	144	–11	–80	12	32.34
Posterior cingulate cortex	L	15	118	–13	–71	39	19.20
Paracentral lobular and mid cingulate cortex	L	37	143	–17	–41	56	24.67
	R	36	129	–7	–39	62	24.90
Superior parietal cortex	L	48	103	–30	–50	48	38.12
	R	48	264	26	–57	47	30.68
Orbital and polar frontal cortex	R	66	120	35	25	-18	21.41

*Statistically significant differences in cortical thickness were defined as P < 0.05 (P < 0.025 for each hemisphere), Monte Carlo Simulation corrected after correcting for age, sex, education, and the head motion. MNI, Montreal Neurological Institute; HCP, Human Connectome Project.*

### *Post hoc* Analyses

**FIGURE 2:**
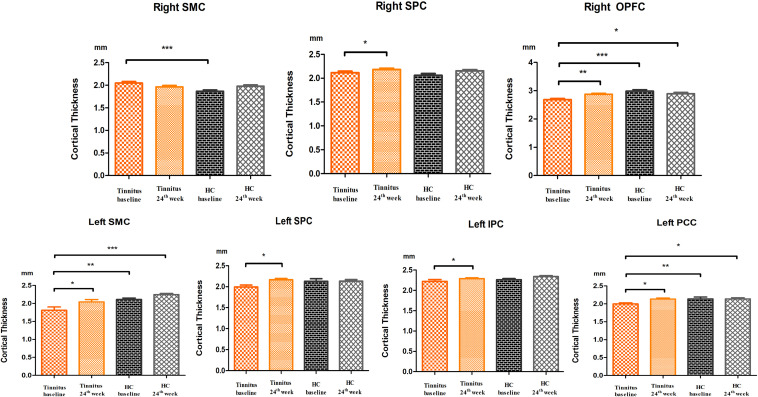
*Post hoc* analyses showed differences in cortical thickness among the baseline and 24-week sound treatment and the HC baseline and HC 24 weeks groups in both hemispheres. *P < 0.05, **P < 0.01, ***P < 0.001. SMC, somatosensory and motor cortex; SPC, superior parietal cortex; PVC, primary visual cortex; PCC, posterior cingulate cortex; IPC, inferior parietal cortex; OPFC, orbital and polar frontal cortex.

Significantly lower cortical thickness was found in the left SMC, left PCC, and right OPFC in the tinnitus group at baseline than in the HC group at baseline and after 24 weeks (Figure 2).

In the tinnitus group, significantly higher cortical thickness was found after the 24 weeks sound therapy in comparison to the baseline in the left SMC, bilateral SPC, left IPC, left PCC, and right OPFC. In this comparison, the difference in the right SMC did not reach statistical significance (Figure 2).

In the HC group, no statistically significant difference in cortical thickness was found after the 24 weeks sound therapy in comparison to the baseline in the bilateral SMC, bilateral SPC, left IPC, left PCC, or right OPFC (Figure 2).

Compared with the HC baseline group and the HC 24 weeks group, the 24 weeks sound therapy tinnitus group showed slightly lower or similar cortical thickness in the bilateral SMC, bilateral SPC, right OPFC, left IPC, and left PCC, and these differences did not reach statistical significance (Figure 2).

In *post hoc* analysis, there were no significant differences between the tinnitus baseline group and the 24 weeks sound therapy tinnitus group in the bilateral PCL and MCC, bilateral DSVC, or left PVC.

#### Correlation

Decreased THI scores and cortical thickness changes in the above brain regions were not significantly correlated.

## Discussion

This is a meaningful SBM investigation that analyzed changes in cortical thickness in tinnitus patients from baseline to after 24 weeks of sound therapy. Cortical thickness changes in the brain were found in patients after longer-term sound therapy, mainly in the left SMC, bilateral SPC, left IPC, left PCC, and right OPFC. These brain regions are mainly concentrated in the visual cortex, prefrontal cortex, somatosensory cortex, motor cortex, and default-mode network (DMN), which are closely related to the abnormal brain morphology associated with tinnitus. Our data confirmed our hypothesis that sound therapy can gradually return the brain structure to a relatively normal range, especially cortical thickness.

In our study, we observed a significant increase in cortical thickness in the left SMC, bilateral SPC, left IPC, left PCC, and right OPFC in the patients after treatment compared with baseline. Among the above brain regions, the left PCC, right medial prefrontal cortex (mPFC), and left IPC are parts of the DMN. DMN is a brain system involved in internal cognitive patterns, and cognitive dysfunction may be linked with neuropathological changes in tinnitus (Vanneste et al., 2016; Wang et al., 2018). The PCC, as the central hub, is a metabolically active and highly connected region in the brain (Fransson and Marrelec, 2008) and plays a key role in stimulus-independent processing (Wei et al., 2020a). Chen et al. (2018a) reported that increased functional connectivity (FC) patterns of the precuneus and inferior parietal lobule (IPL) might be responsible for disrupting the DMN in tinnitus patients. Chen et al. (2018b) also showed that decreased FC within the DMN was associated with poorer cognitive performance in chronic tinnitus patients. This shows that tinnitus has a very close relationship with DMN.

The orbitofrontal cortex and PFC constitute the 20th section of the HCP template—the OPFC (Glasser et al., 2016). The orbitofrontal cortex is a large but heterogeneous cortical area on the ventral surface of the frontal lobe and has been closely related to emotion and executive function (Rudebeck and Rich, 2018). The PFC is involved in the executive control of attention and tinnitus perception (Aldhafeeri et al., 2012; Schmidt et al., 2013). Cortical thickness reductions and disrupted connectivity of the PFC might also be associated with the negative emotions experienced by tinnitus sufferers (Aldhafeeri et al., 2012). The IPC is also involved in the combination of auditory, visual, somatosensory, and attention processes. The results of our study indicated that lower DMN cortical thickness correlated with performance in the above domains in tinnitus patients, and our results are more focused on explaining morphological changes. In addition to DMN, the superior parietal lobule (SPL) is also involved in the cognitive process, and early along the auditory pathway, different cognitive resources are required for the filtering out of tinnitus (Vanneste et al., 2018). After sound therapy, the cortical thickness in these brain regions was slightly increased, and whether their corresponding functions are restored still needs further research.

The precentral gyrus (PreCG) is a typical representative of the somatosensory and primary motor cortex from the HCP brain template, and it plays a role in auditory-associated functions (Zhang et al., 2015; Glasser et al., 2016). Some studies have reported that the PreCG might associate with word recognition and phonological processing (Xu et al., 2019). Another study found significant functional changes between the PreCG and some other regions in tinnitus patients and inferred that the changes may suggest that tinnitus was caused or modulated by signals from the somatosensory, somatomotor, and visuomotor systems in some patients (Feng et al., 2018). Our results also indicated that sound therapy can affect cortical thickness at the morphological level. Although the change in the PreCG may not be as significant as functional changes, the results also reflect the efficacy of sound therapy in altering SBM properties to a certain extent.

In our *post hoc* analysis, there were no significant differences in the bilateral PCL and MCC, bilateral DSVC, or right PVC, but there were group differences. The PCL and MCC are included in the HCP template, and the former is adjacent to the somatosensory area (SMA), which was proposed to be involved in sensorimotor integration (Edwards et al., 2019; Umeda et al., 2019). The DSVC and PVC are visual cortexes. Some studies have reported that auditory spatial information is processed by the visual centers of the brain in addition to auditory brain regions (Bedny et al., 2010; Fiehler and Rosler, 2010). A recent study reported increased activity in the right middle occipital gyrus in tinnitus patients; the middle occipital gyrus is associated with visual processing, which may process cross-modal information in phantom auditory perception (Cheng et al., 2020). These brain regions are also irreplaceable neuroimaging targets in assessing tinnitus treatment efficacy.

Although different studies have used different morphological analysis methods, the correct treatment method and the duration of treatment are the most critical factors for achieving therapeutic effects. Schmidt et al. (2018) used SBM to investigate GM changes and diffusion tensor imaging to assess changes in orientation of WM tracts and found that whole-brain analyses revealed decreased cortical thickness in the left parahippocampal gyrus in those with more severe tinnitus compared to a group with a milder reaction. However, the individuals in that study were not being treated. A previous study with the Heidelberg model of music therapy analyzed the average treatment time to be approximately 2 weeks (Krick et al., 2015) and found GM volume changes in the precuneus, medial superior frontal areas, and auditory cortex. In our study, we applied narrowband noise sound therapy over a relatively long (6 months) treatment time. The morphological changes that may be found with this type of treatment and duration of treatment are different from those of previous studies, which is supported by our results. Our previous study showed that FC of the right insula, IPL, bilateral thalami, and left middle temporal gyrus was significantly modified with sound and predicted the clinical outcome of adjusted narrowband noise sound therapy (Han et al., 2019a). As in our former study (Lv et al., 2020) proved, from a functional perspective, the function of the brain regions mentioned above were basically improved after sound therapy. The results of this study are morphologically proven after a longer-term treatment, and the cortical thickness in these brain regions was slightly increased.

## Limitations

First, there was no significant relationship between ΔTHI scores and cortical thickness changes in tinnitus patients, which may be due to the small sample size. In future studies, we need to further expand the sample size to verify the correlation between them. Second, due to the huge amount of high-precision cortical reconstruction calculations, the preprocessing time of SBM is longer; therefore, no studies have reported the treatment effect of tinnitus from the perspective of SBM. In future research, we need to use a more computationally intensive computer to analyze the morphology of tinnitus from all dimensions of SBM, such as cortical volume, curvature, cortical areas, and sulcal depth. Lastly, the current findings should not be overgeneralized to the entire tinnitus population as sound therapy is not the only possible treatment for tinnitus patients, and measurable tinnitus pitch was included in this study, which did not account for patients who reported other tinnitus sounds or characteristics.

## Conclusion

This study analyzed anatomical changes in tinnitus patients before and after treatment for 6 months. Sound therapy can change cortical thickness in some brain regions and systems that are important for SMC, superior parietal cortex, inferior parietal cortex, PCC, and OPFC. Surface-based morphometry to assess cortical thickness can potentially provide neuroimaging research value to the study of tinnitus-related neuroanatomical changes before and after sound therapy in tinnitus patients. The findings of this study may better support the reduction in tinnitus severity or increase in cortical thickness.

## Data Availability Statement

The original contributions presented in the study are included in the article/supplementary material, further inquiries can be directed to the corresponding author/s.

## Ethics Statement

The studies involving human participants were reviewed and approved by the Ethics Committees of our Research Institution (Beijing Friendship Hospital, Capital Medical University, 2016-P2-012). The patients/participants provided their written informed consent to participate in this study. Written informed consent was obtained from the individual(s) for the publication of any potentially identifiable images or data included in this article.

## Author Contributions

XW designed the experiments, performed the statistical analysis, and wrote the manuscript. QC conducted the statistical analysis. PZ contributed to the manuscript revision. HL, ZW, and CL collected the data. SG, ZY, and ZW were guarantors of this work. HL and ZW were the corresponding authors of this manuscript, full access to all the data in the study, take responsibility for the integrity of the data, and the accuracy of the data analysis. All authors contributed to the article and approved the submitted version.

## Conflict of Interest

The authors declare that the research was conducted in the absence of any commercial or financial relationships that could be construed as a potential conflict of interest.

## Footnotes

1 http://rfmri.org/DPABISurf
